# Emerging Threat of Antimicrobial Resistance in β-Hemolytic Streptococci

**DOI:** 10.3389/fmicb.2020.00797

**Published:** 2020-05-15

**Authors:** Oddvar Oppegaard, Steinar Skrede, Haima Mylvaganam, Bård Reiakvam Kittang

**Affiliations:** ^1^Department of Medicine, Haukeland University Hospital, Bergen, Norway; ^2^Department of Clinical Science, Faculty of Medicine, University of Bergen, Bergen, Norway; ^3^Department of Microbiology, Haukeland University Hospital, Bergen, Norway; ^4^Department of Medicine, Haraldsplass Deaconess Hospital, Bergen, Norway

**Keywords:** β-hemolytic streptococci, antimicrobial resistance, *Streptococcus dysgalactiae*, *Streptococcus pyogenes*, *Streptococcus agalactiae*

## Abstract

Highly variable resistance rates to erythromycin and clindamycin have been reported in the β-hemolytic streptococcal species *Streptococcus pyogenes*, *Streptococcus agalactiae*, and *Streptococcus dysgalactiae*, depending on geographic and temporal context. In the present study we aimed to examine the longitudinal trends of antimicrobial resistance in these three species in a northern European setting. Furthermore, we used whole genome sequencing to identify resistance determinants and the mobile genetic elements involved in their dissemination, as well as elucidate phylogenetic relationships. All cases of invasive β-hemolytic streptococcal diseases in Health Region Bergen, western Norway, in the period 2004 to 2018 were retrospectively identified, comprising 271, 358, and 280 cases of *S. pyogenes*, *S. agalactiae*, and *S. dysgalactiae*, respectively. Antimicrobial susceptibility testing revealed a gradual but significant increase in erythromycin and clindamycin resistance for *S. agalactiae* and *S. dysgalactiae* during the study period. Whole genome sequencing of the erythromycin and clindamycin resistant bacterial population revealed a substantial phylogenetic diversity in *S. agalactiae* and *S. dysgalactiae*. However, the mobile genetic elements harboring the resistance determinants showed remarkable intra- and interspecies similarities, suggesting a dissemination of antimicrobial resistance predominantly through conjugative transfer rather than clonal expansion of resistant strains in these two species. Conversely, antimicrobial resistance in *S. pyogenes* remained low, apart from a transient outbreak of a clindamycin and erythromycin resistant *emm11*/ST403-clone in 2010–2012. Increased epidemiological attentiveness is warranted to monitor the emerging threat of antimicrobial resistance in β-hemolytic streptococci, particularly in *S. agalactiae* and *S. dysgalactiae*.

## Introduction

The major human pathogens among β-hemolytic streptococci (BHS) are *Streptococcus pyogenes* (Lancefield group A streptococcus, GAS), *Streptococcus agalactiae* (Lancefield group B streptococcus, GBS), and *Streptococcus dysgalactiae* (Lancefield group C and G streptococcus, SD). These phylogenetically closely related species produce overlapping clinical manifestations, and collectively they are responsible for substantial global disease burden (Carapetis et al., 2005; Le Doare and Heath, 2013; Rantala, 2014). Historically, SD was regarded as a rare cause of human disease, but the past decades a rapidly increasing incidence of invasive SD infections has been documented, and the rates have surpassed those of GAS and GBS in several geographic regions (Bramhachari et al., 2010; Oppegaard et al., 2015; Wajima et al., 2016).

Although Penicillin remains the drug of choice for treating infections caused by BHS, macrolides and clindamycin are important alternatives in β-lactam-intolerant patients. Furthermore, adjunctive clindamycin therapy reduces mortality in patients suffering from severe GAS disease manifestations (Linner et al., 2014), probably through the abrogation of bacterial toxin production (Mascini et al., 2001).

Resistance to the antimicrobial classes macrolides, lincosamides (such as clindamycin) and streptogramin B (MLS_*B*_) in BHS is primarily linked to the acquisition of target modification enzymes encoded by *erm* genes, mediating resistance to all three classes, or *mef* genes encoding efflux pumps targeting only macrolides (Varaldo et al., 2009). The genes *lsa* and *lnu*, causing lincosamide resistance, are also occasionally encountered (Hawkins et al., 2017). In GAS and GBS, all these resistance genes are associated with mobile genetic elements, and dissemination of these elements occur by either clonal expansion or horizontal genetic transfer (Varaldo et al., 2009; Palmieri et al., 2013; Hawkins et al., 2017; Zhou et al., 2017). The genetic environment of resistance determinants in SD is largely unknown, and the mechanisms involved in their dissemination have not been extensively explored. However, *in vitro* conjugal transfer of integrative conjugative elements (ICEs) harboring resistance genes has been demonstrated between these three species, and a common reservoir for resistance determinants is feasible (Palmieri et al., 2013).

There are considerable variations in reported resistance rates to MLS_*B*_ antibiotics in BHS, and differences in susceptibility-testing methodology and ever-changing reference breakpoints make temporospatial comparisons difficult. Antimicrobial susceptibility rates in both GAS and GBS are under national surveillance in some countries, and whereas resistance to erythromycin and clindamycin appears to have a fluctuating course among GAS, gradually increasing rates of resistance has been noted in GBS (Norm/Norm-Vet, 2018; Public Health England, 2018; Swedres-Swarm, 2018) currently approximating 50% for both agents in the United States of America^1^. Consequently, the Infectious Diseases Society of America recently revised their national guidelines for antimicrobial treatment of GBS-infections (CDCgov, 2010). Long term epidemiologic trends of resistance rates in SD have not been previously studied in detail.

In the present study, we sought to examine the temporal trends of antimicrobial resistance in invasive BHS in western Norway, with a particular emphasis on SD. Moreover, we used whole genome sequencing to identify validated resistance genes and characterize their associated mobile genetic elements, as well as explore phylogenetic relationships and clonality in the resistant bacterial population.

## Materials and Methods

### Study Setting and Definitions

Health Region Bergen in western Norway has a catchment area of approximately 450,000 inhabitants, and comprises the tertiary care hospital Haukeland University Hospital, along with the two secondary care hospitals Haraldsplass Deaconess Hospital and Voss Hospital. All cases of invasive β-hemolytic streptococcal infections in this region presenting in the period 2004–2018 were retrospectively identified. Invasive disease was defined as isolation of BHS from normally sterile sites or from a non-sterile site in combination with surgically proven necrotizing soft tissue infection. Culture negative cases identified by sequencing of the 16S rRNA gene were excluded. To avoid issues regarding persistent and relapsing infections, only one incident per person was included.

### Bacterial Isolates

All BHS have been identified based on large colony size (>0.5 mm in diameter) and β-hemolytic reaction on 5% sheep blood agar after incubation for 24 h, along with serogroup specificity using a rapid agglutination test (Oxoid, Basingstoke, Hampshire, United Kingdom). Species identity has been confirmed using Matrix Assisted Laser Desorption Ionization Time of Flight Mass Spectrometry (MALDI ToF MS) with the MALDI Biotyper database (Bruker Daltonik, Bremen, Germany).

### Antimicrobial Susceptibility Testing

The isolates were tested for susceptibility to penicillin G, erythromycin, clindamycin, tetracycline, and trimethoprim-sulfamethoxazole by the disk diffusion method in accordance with the European Committee on Antimicrobial Susceptibility Testing (EUCAST) guidelines^2^. MLS_*B*_ resistance phenotype was tested using the double disc diffusion method (D-test), placing erythromycin and clindamycin discs edge to edge 12 mm apart. Isolates resistant to erythromycin and/or clindamycin were categorized as having constitutive MLS_*B*_-resistance (cMLS_*B*_), inducible MLS_*B*_-resistance (iMLS_*B*_), macrolide resistance alone (M-phenotype), or isolated lincosamide resistance (L-phenotype). *Streptococcus pneumoniae* ATCC 49619 was used as control strain. EUCAST clinical breakpoints version 9.0 (2019) were used for categorization into susceptible, susceptible increased exposure and resistant phenotypes (see text footnote 2).

### Whole Genome Sequencing and Bioinformatics

All isolates displaying reduced susceptibility to erythromycin or clindamycin were subjected to whole genome sequencing. Bacterial DNA was extracted using MagNA Pure (Roche Life science, Basel, Switzerland) and quantified using Qubit (Thermo Fisher Scientific, United States). Genomic libraries were constructed using the Illumina Nextera XT kit (Illumina, Essex, United Kingdom), and 150 base pair paired end sequencing was performed on a MiSeq platform (Illumina).

Paired end reads were assessed for quality using FastQC^3^, trimmed with Trimmomatic (Bolger et al., 2014), assembled by Spades (Nurk et al., 2013), and subsequently annotated using RAST (Aziz et al., 2008).

The *emm*-types of GAS and SD isolates were verified in-silico by BLAST-search against the *emm*-type database^4^. Capsule-types in GBS isolates were also checked using a pipeline developed by Metcalf (Metcalf et al., 2017). Multilocus sequence typing (MLST) profiles were identified using the Center for Genomic Epidemiology (CGE) website (Larsen et al., 2012).

Relevant resistance genes were identified by BLAST-searches in Geneious (Kearse et al., 2012), using the ResFinder and CARD databases as references (Zankari et al., 2012; Alcock et al., 2020). The genomic context of any identified resistance determinants was subsequently manually explored in Geneious for the characterization of their association with mobile genetic elements. Verification of potential ICEs and bacteriophages was performed using ICEfinder (Liu et al., 2019) and PHASTER (Arndt et al., 2016), in combination with meticulous literature review and BLAST-searches. Mauve was used for comparison of ICEs (Darling et al., 2004).

Phylogenetic relationships and clonality among the resistant isolates were evaluated by analysis of single nucleotide polymorphisms (SNPs) using CSI Phylogeny version 1.4 on the CGE website at default settings. One resistant isolate for each species were randomly picked as reference genome. Phylogenetic trees were constructed in Geneious using Geneious tree builder with the Tamura-Nei Genetic distance model and Neighbor-joining method without bootstrapping.

### Statistics

Temporal trends were evaluated for statistical significance by Poisson regression analysis. Data were analyzed using SPSS PASW STATISTICS, version 23.0 (IBM SPSS Statistics for Windows, Armonk, NY, United States: IBM Corp.). A two-tailed *p*-value <0.05 was considered significant.

### Ethical Statement

The study underwent institutional ethics review and approval (2010/1406 Regional Ethics Committee West, Norway). The study was exempted from written consent as it does not involve patient information or human biological material.

## Results

We identified a total of 995 culture positive cases of invasive BHS disease, affecting 957 patients in the period 2004–2018. Recurrent infections were predominantly caused by GBS and SD (17 cases each). Clinical isolates were available for antimicrobial susceptibility testing for 909 out of the 957 unique cases (95%), comprising 271 GAS, 358 GBS, and 280 SD.

### Antimicrobial Susceptibility

All isolates were fully susceptible to penicillin G, and only one isolate each of GAS and SD displayed phenotypic resistance to trimethoprim-sulfamethoxazole. Reduced susceptibility to tetracycline was observed in 14%, 67%, and 40% of GAS, GBS, and SD isolates, respectively. A total of 87 isolates were resistant to MLS_*B*_ antibiotics, comprising 15 GAS, 53 GBS, and 19 SD. The distribution of the zone diameters for all the tested antimicrobial agents is presented in Table 1.

**TABLE 1:** Distribution of antimicrobial resistance testing zone diameters.

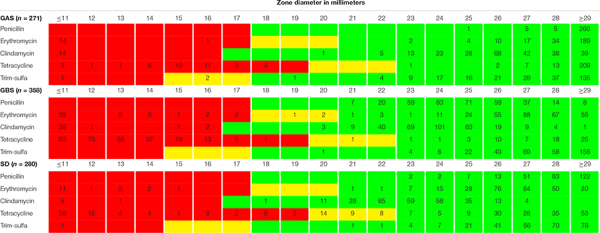

*The distribution is presented as the number (*n*) of isolates in each zone diameter category. Isolates have been categorized as susceptible (green), susceptible increased exposure (yellow), or resistant (red) using EUCAST breakpoints v9.0 (2019). GAS, *Streptococcus pyogenes*; GBS, *Streptococcus agalactiae*; SD, *Streptococcus dysgalactiae*; Trim-sulfa, trimethoprim-sulfamethoxazole.*

**FIGURE 1:**
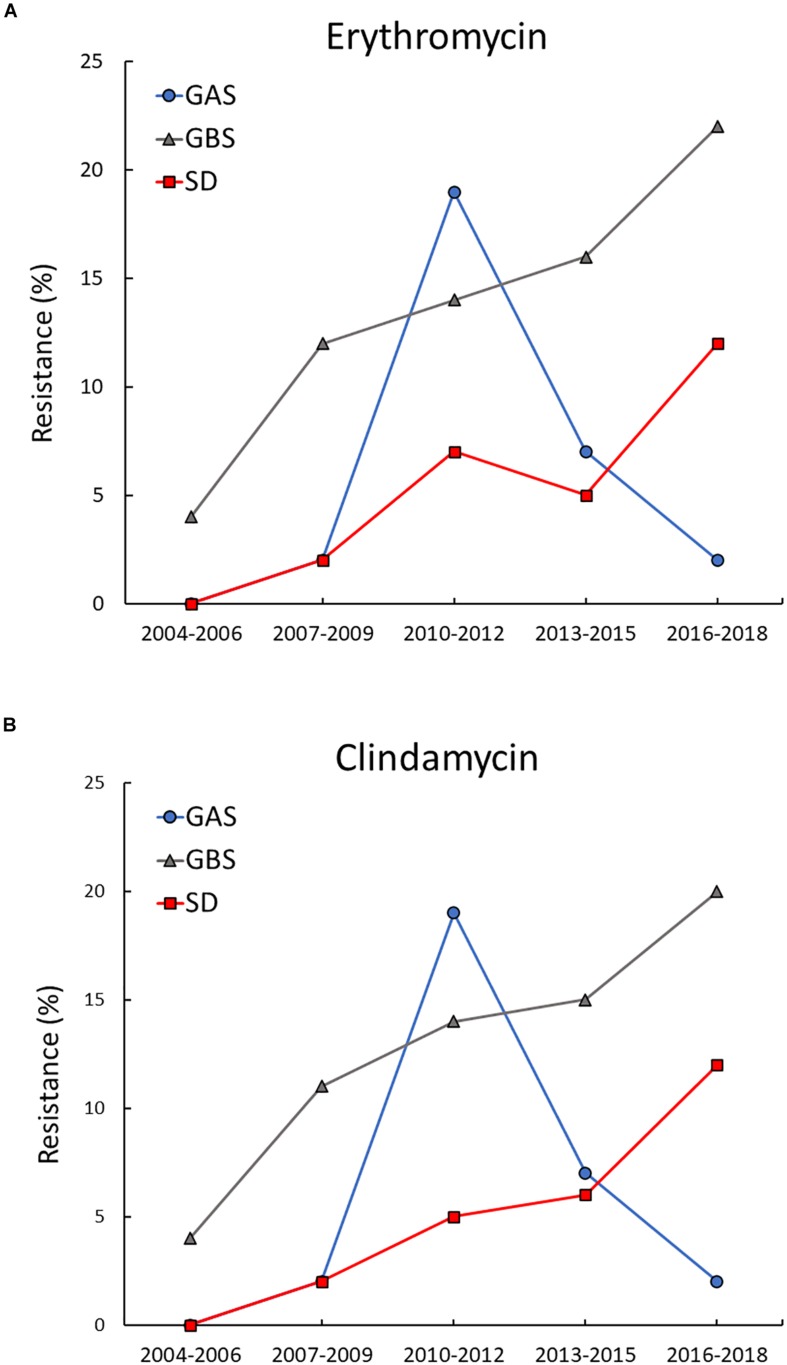
Temporal trends of erythromycin and clindamycin resistance in BHS. Resistance to erythromycin **(A)** and clindamycin **(B)** increased significantly in *Streptococcus agalactiae* (GBS) and *Streptococcus dysgalactiae* (SD) during the study period. In *Streptococcus pyogenes* (GAS) resistance to erythromycin and clindamycin remained low, apart from a transient outbreak of resistance to both agents in the period 2010–2012.

Distinct temporal trends for erythromycin and clindamycin resistance were revealed for the three BHS species (Figure 1). For GAS, the overall prevalence of resistance was very low, apart from a transient increase in resistance to erythromycin and clindamycin in 2010–2012. During this period almost 20% of the invasive GAS isolates belonged to a cMLS_*B*_-resistant *emm11*-lineage, but the outbreak quickly abated. The rates of resistance in GBS displayed a gradual and significant increase in the study period, rising from 4% during 2004–2006 to 22% in the period 2016–2018 for erythromycin (*p* = 0.004), and from 4 to 20% for clindamycin (*p* = 0.013). A similar upward trend was observed for SD, where no resistant isolates where detected until 2009. Subsequently, the incidence of erythromycin and clindamycin resistance rose significantly to 12% for both agents by 2016–2018 (*p* = 0.012).

### Detection of Resistance Genes

All 87 BHS isolates displaying reduced susceptibility to MLS_*B*_ antibiotics were subjected to whole genome sequencing, and in 86 of these validated resistance genes were detected (Table 2). Chromosomal mutations in ribosomal genes were not investigated. Almost all isolates displaying the iMLS_*B*_ phenotype harbored the *erm*(A)-gene, but *erm*(T) was found in one GBS isolate. The cMLS_*B*_ phenotype was predominantly associated with the *erm*(B)-gene, *mef*(A) was linked to the M phenotype and the *lsa*(C)-gene was present in three isolates with the L phenotype. Presence of additional clindamycin-resistance genes was found in three *erm*(B)-positive GBS isolates; one harbored both *lsa*(E) and *lnu*(B), and in two isolates *lsa*(C) was detected.

**TABLE 2:** Antimicrobial resistance phenotype and genotype of the macrolide and lincosamide resistant β-hemolytic streptococcal isolates.

Microbe	*n*	Phenotype	(*n*)	Genotype (*n*)
*S. pyogenes*	15	iMLS_*B*_	(1)	*erm*(A) (1)
		cMLS_*B*_	(14)	*erm*(B) (14)
		M	(0)	
		L	(0)	
		Tet R	(14)	*tet(M)* (14)
*S. agalactiae*	53	iMLS_*B*_	(7)	*erm*(A) (6), *erm*(T) (1)
		cMLS_*B*_	(38)	*erm*(A) (8), *erm*(B) (26), *erm*(B) + *lsa*(C) (2), *erm*(B) + *lsa*(E) + *lnu*(B) (1), ND (1)
		M	(6)	*mef*(A) (6)
		L	(2)	*lsa*(C) (2)
		Tet R	(50)	*tet*(M) (31), *tet*(O) (17), *tet*(M) + *tet*(O) (2)
*S. dysgalactiae*	19	iMLS_*B*_	(11)	*erm(*A) (11)
		cMLS_*B*_	(6)	*erm*(A) (3), *erm*(B) (3)
		M	(1)	*mef*(A) (1)
		L	(1)	*lsa*(C) (1)
		Tet R	(6)	*tet*(M) (4), *tet*(O) (1), *tet*(M) + *tet*(O) (1)

**n*, number of isolates; MLS_*B*_, macrolide, lincosamide, streptogramin B; iMLS_*B*_, inducible MLS_*B*_-resistance; cMLS_*B*_, constitutive MLS_*B*_-resistance, M, macrolide resistance alone; L, lincosamide resistance alone; Tet R, tetracycline resistance; ND, no resistance gene detected.*

Co-resistance to tetracycline was evident in almost all GAS (14/15) and GBS (50/53) with reduced susceptibility to MLS_*B*_ antibiotics, whereas only 31% (6/19) of the SD isolates displayed dual resistance. *tet*(M) was the most common resistance determinant in all three species, but *tet*(O) was also frequent in GBS (Table 2).

The 14 cMLS_*B*_ and tetracycline resistant GAS isolates were discovered also to encode a cassette of genes mediating resistance to aminoglycosides and streptothricins, comprising *aad*E, *aph*A3, and *sat*4. A similar element was detected in 14 cMLS_*B*_ and tetracycline resistant GBS isolates. The single GAS and SD isolates resistant to trimethoprim-sulfamethoxazole were also whole genome sequenced and found to contain the *dfr*(A)-gene.

### Phylogenetic Analysis of the BHS Population Resistant to Erythromycin and Clindamycin

To facilitate temporospatial comparisons of clonal distribution of macrolide and clindamycin resistance, an analysis of the combination of *emm*-type, MLST-type and MLS_*B*_-resistance gene has often been used as a rough marker for resistant clones in GAS (Perez-Trallero et al., 2007; Willems et al., 2011). In the present study we have extrapolated this approach to SD, and substituted *emm*-type with capsule-type for GBS. An overview of the spectrum of resistant clones for the three species is presented in Table 3.

**TABLE 3:** Genetic characterization of the resistant BHS population.

	Clone		*n*	Phenotype	ICE-family	Ref	Other resistance genes
	GAS						
	
*emm*	*MLST*	*res-gene*					

*emm11*	ST403	*erm*(B)	14	cMLS_*B*_	Tn*6003*	A	*tet*(M) (14), MAS (14)
*emm58*	ST176	*erm*(A)	1	iMLS_*B*_	ICE*sp2905*	B	
	GBS						
	
*Capsule*	*MLST*	*res-gene*					

Ib	ST12	*erm*(B)	3	cMLS_*B*_	ICE*Sa2603*	C	*tet*(O) (3), MAS (3), *lsa*(E) + *lnu*(B) (1)
II	ST1	*erm*(A)	1	iMLS_*B*_	Unknown		*tet*(M)
II	ST12	*erm*(B)	1	cMLS_*B*_	ICE*Sa2603*	C	*tet*(O)
II	ST22	*erm*(B)	1	cMLS_*B*_	Tn*3872*	A	*tet*(M)
III	ST17	*erm*(B)	9	cMLS_*B*_	ICE*Sa2603*	C	*tet*(O) (9), *tet*(M) (2), MAS (8)
III	ST1434	*erm*(B)	1	cMLS_*B*_	ICE*Sa2603*	C	*tet*(O), MAS
III	ST19	*erm*(B)	4	cMLS_*B*_	ICE*Sa2603*	C	*tet*(O) (2), *tet*(M) (2), *lsa*(C) (2), MAS (2)
III	ST27	*erm*(B)	1	cMLS_*B*_	ICE*Sa2603*	C	*tet*(O)
III	ST1432	*erm*(B)	1	cMLS_*B*_	Tn*3872*	A	*tet*(M), *cat*(p*c194*)
III	ST335	*erm*(A)	2	iMLS_*B*_	ICE*sp2905*	B	*tet*(M) (2)
III	ST107	*erm*(A)	1	cMLS_*B*_	ICE*sp2905*	B	
IV	ST1433	*erm*(B)	1	cMLS_*B*_	ICE*Sa2603*	C	*tet*(O)
IV	ST459	*erm*(A)	6	cMLS_*B*_	ICE*sp2905*	B	*tet*(M) (5)
V	ST1	*erm*(A)	4	iMLS_*B*_	Unknown		*tet*(M) (4)
V	ST1	*erm*(B)	7	cMLS_*B*_	Tn*3872*	A	*tet*(M) (7)
III	ST17	*erm*(T)	1	iMLS_*B*_	p*RW35*	A	*tet*(M)
Ia	ST23	*mef*	6	M	Phage1207.3	A	*tet*(M) (6)
III	ST1399	*lsa*(C)	1	L	IME*SagUCN70*	D	*tet*(M)
III	ST1167	*lsa*(C)	1	L	IME*SagUCN70*	D	*tet*(O)
Ia	ST7	*ND*	1	cMLS_*B*_	ND		
	SD						
	
*emm*	*MLST*	*res-gene*					

*stC74a*	ST17	*erm*(A)	4	iMLS_*B*_	ICE*sp2905*	B	
*stC74a*	ST29	*erm*(A)	1	iMLS_*B*_	ICE*sp2905*	B	*tet*(M)
*stG480*	ST8	*erm*(A)	2	iMLS_*B*_	ICE*sp2905*	B	
*stG245*	ST29	*erm*(A)	1	cMLS_*B*_	ICE*sp2905*	B	
*stG62647*	ST319	*erm*(A)	1	iMLS_*B*_	ICE*sp2905*	B	
*stG2574*	ST23	*erm*(A)	1	cMLS_*B*_	ICE*sp2905*	B	*tet*(M)
*stG485*	ST55	*erm*(A)	1	iMLS_*B*_	ICE*sp2905*	B	*tet*(M) + *tet*(O)
*stG485*	ST17	*erm*(A)	1	cMLS_*B*_	ICE*sp2905*	B	
*stG485*	ST29	*erm*(B)	1	cMLS_*B*_	Unknown		
*stG2078*	ST473	*erm*(A)	1	iMLS_*B*_	ICE*sp2905*	B	
*stG2078*	ST17	*erm*(A)	1	iMLS_*B*_	ICE*sp2905*	B	
*stG2078*	ST17	*erm*(B)	1	cMLS_*B*_	Tn*6218*	*	
*stG840*	ST269	*erm*(B)	1	cMLS_*B*_	Tn*3872*	A	*tet*(M)
*stG485*	ST128	*mef*(A)	1	M	Phage1207.3	A	*tet*(M)
*stG643*	ST12	*lsa*(C)	1	L	ICE*sp2905*	B	*tet*(O)

A (Varaldo et al., 2009); B (Giovanetti et al., 2012); C (Hawkins et al., 2017), and D (Zhou et al., 2017). *Unpublished, GenBank accession HG002388. *n*, number of isolates; ICE, integrative conjugative element; MLST, multilocus sequence typing; res-gene, resistance gene; GAS, *Streptococcus pyogenes*; GBS, *Streptococcus agalactiae*; SD, *Streptococcus dysgalactiae*; MLS_*B*_, macrolide, lincosamide, streptogramin B; iMLS_*B*_, inducible MLS_*B*_-resistance; cMLS_*B*_, constitutive MLS_*B*_-resistance, M, macrolide resistance alone; L, lincosamide resistance alone; ND, no resistance gene detected. MAS, macrolide-aminoglycoside-streptothricin resistance element containing the aminoglycoside and streptothricin resistance genes *aadE*, *aphA3*, and *sat4* in addition to the macrolide resistance determinant *ermB*.

For GAS, the *emm11*/ST403/*erm*(B) lineage dominated, and only one isolate was found to belong to a different clade, *emm58*/ST176/*erm*(A). Differently, 20 different clones were identified in resistant GBS isolates, the predominant being III/ST17/*erm*(B) (*n =* 9), V/ST1/*erm*(B) (*n =* 7), IV/ST459/*erm*(A) (*n =* 6), and Ia/ST23/*mef*(A) (*n =* 6). A similar diversity was observed in SD, where 15 different lineages were observed among the 19 isolates resistant to MLS_*B*_ antibiotics. Only *stC74a*/ST17/*erm*(A) (*n =* 4) and *stG480*/ST8/*erm*(A) (*n =* 2) were associated with more than one isolate. All sequenced SD isolates belonged to the subspecies *S. dysgalactiae* subspecies *equisimilis*.

**FIGURE 2:**
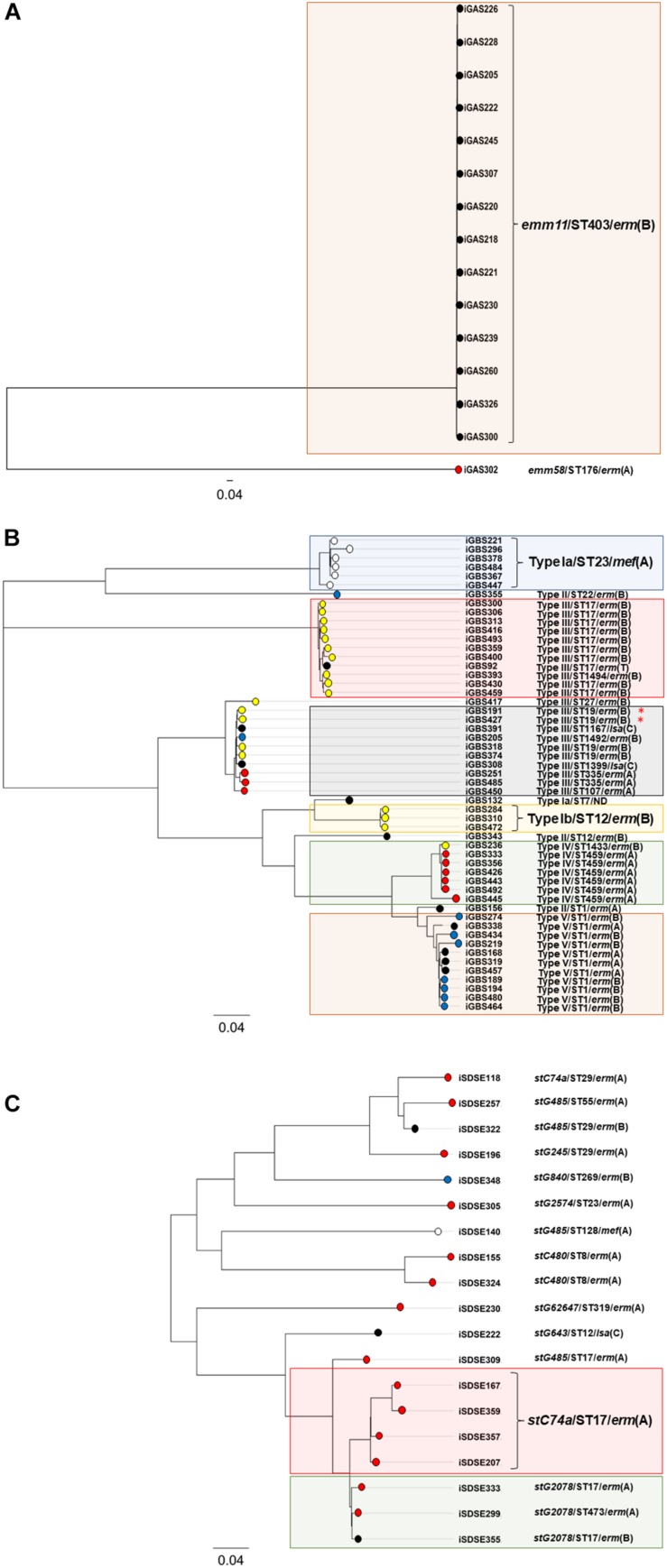
Phylogenetic trees of the resistant BHS – population. The phylogenetic trees are based on core-genome SNP-analysis of *S. pyogenes* **(A)**, *S. agalactiae* **(B)**, and *S. dysgalactiae* **(C)** isolates displaying reduced susceptibility to erythromycin and/or clindamycin. The scale indicates substitutions per site. Isolates clustering with >97% similarity have been highlighted with colored rectangular boxes. The circular node tips have been assigned color coding according to the mobile genetic element harboring the MLS_*B*_-resistance gene in the respective isolates. Red indicates ICE*sp2907*, blue color has been assigned to Tn*3872*, yellow indicates ICE*Sa2603*, isolates carrying the bacteriophage 1207.3 are white, and all other elements are depicted in black. The red asterisk indicates the two isolates co-harboring *lsa*(C).

Phylogeny of the erythromycin and clindamycin resistant BHS population was also examined by SNP-analysis. The isolates iGAS300, iGBS300, and iSDSE357 were randomly chosen as reference genomes, yielding a core genome size of 1.7 Mbp (94% of reference genome), 1.7 Mbp (82%), and 1.8 Mbp (83%) for GAS, GBS, and SD, respectively. In GAS, 7,550 valid SNP-positions were found, and the isolates differed by a range from 4 to 7,528 SNPs. However, the isolates belonging to the *emm11*/ST403/*erm*(B) lineage were only separated by a maximum of 18 SNPs, indicating a highly clonal group. A total of 19,645 valid SNPs was detected in GBS, ranging from 3 to 8,979 SNP variations between the isolates, whereas in SD 15,524 SNPs were found (range 82–5,865). Phylogenetic trees based on the SNP-analysis are depicted in Figure 2.

### Characterization of Mobile Genetic Elements Associated With Macrolide and Lincosamide Resistance

A wide variety of mobile genetic elements carrying MLS_*B*_-resistance genes were detected in the BHS isolates (Table 3). *mef*(A) was the only resistance gene associated with a bacteriophage (Phage 1207.3), and *erm*(T) was carried by the small plasmid, *pRW35*. The remaining resistance genes were carried by ICEs. The *emm11*/ST403/*erm*(B) GAS lineage was equipped with ICETn*6003*, a composite element derived from the fusion of a multi resistance macrolide, aminoglycoside, streptothricin (MAS) element into *orf20* of the *tet*(M) tetracycline resistance gene carrying Tn*916* element. Tn*3872*, another Tn*916* based element with *erm*(B) integrated in *orf9*, was harbored by nine GBS isolates and one SD isolate. Mobile genetic elements belonging to the ICE*Sa2603* family were predominant among GBS, detected in 20 *erm*(B) positive isolates. Fourteen of these ICE*Sa2603* elements also contained a MAS-like fragment, eighteen carried *tet*(O), whereas two co-harbored *tet*(M).

**FIGURE 3:**
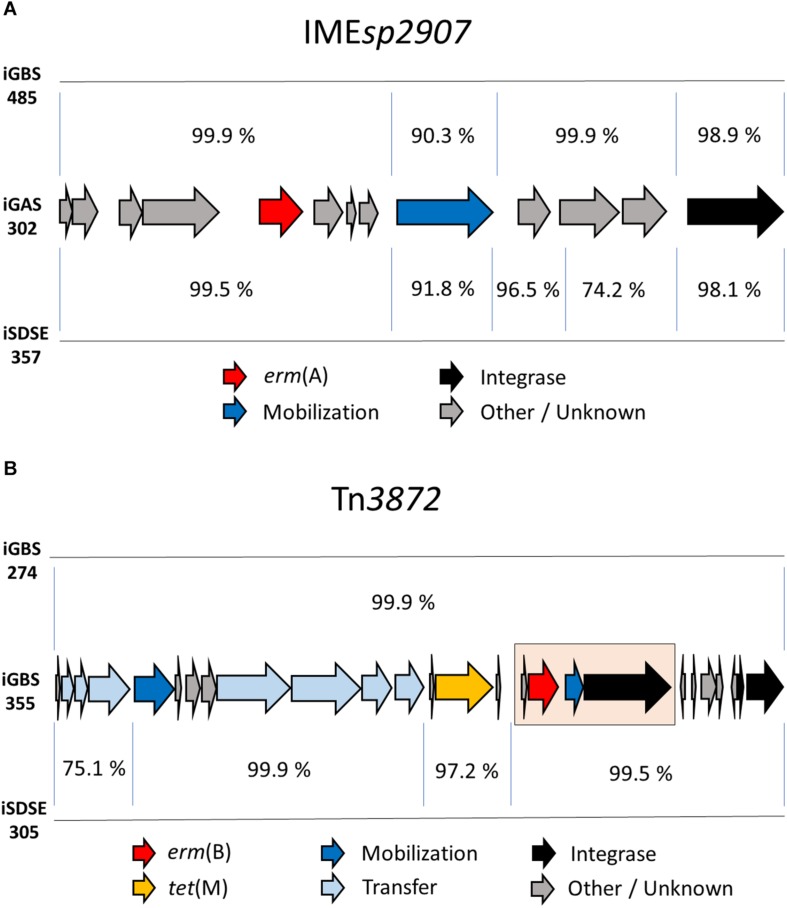
Genetic composition of the mobile genetic element IME*sp2907* in three BHS. Mauve was used for the sequence alignment of the mobile genetic element. Panel **(A)** depicts a comparison of IME*sp2907* detected in the invasive BHS isolates iGAS302 (*S. pyogenes*), iGBS485 (*S. agalactiae*), and iSDSE357 (*S. dysgalactiae*). The integrative mobilizable element displayed a conserved genetic architecture and highly similar nucleotide sequences in these three species. Genes involved in mobilization and integration accounted for the majority of the discrepancies. The gene immediately downstream from *erm*(A) is annotated by RAST as a spectinomycin resistance gene based on sequence similarities, but has not been experimentally verified. Panel **(B)** displays an alignment of Tn*3872* detected in two GBS isolates and one SD isolate. The ICE Tn*3872* is composed of an *erm*(B) module (highlighted in a pink box) integrated into a Tn*916* element.

Interestingly, the composite ICE*sp2905*, with its embedded integrative mobilizable element IME*sp2907* carrying *erm*(A), was detected in all three species, and was the dominant cause of resistance to MLS_*B*_ antibiotics in SD (14/19 isolates). Comparison of IME*sp2907* from different isolates revealed a high degree of similarity (Figure 3A). On a similar note, highly homologous ICEs were found in phylogenetically distantly related GBS isolates, as judged by core-genome SNP-analysis. Although the isolates iGBS310 and iGBS313 differ by 8632 SNPs, they contained ICE*Sa2603* elements displaying 99.5% whole sequence homology (65,877/66,185 bp identity). In the equally phylogenetically distanced isolates iGBS274 and iGBS355, we detected Tn*3872* elements showing 99.9% similarity (23,102/23,113 bp identity) (Figure 3B). Moreover, comparison to the Tn*3872* element in iSDSE305 also revealed a high genetic resemblance (96.1%).

In GBS, all four *lsa*(C) genes were found on the small integrative mobilizable element IME*SagUCN70*, whereas the lincosamide resistance determinants *lsa*(E) and *lnu*(B) co-resided on a mobile genetic element previously described in a GBS isolate (SGB76) (Hawkins et al., 2017). Differently, the *lsa*(C) gene detected in SD was directly integrated into an ICE of the ICE*sp2905*-family.

Two resistance gene related elements, associated with *erm*(A) and *erm*(B), respectively, could not be identified through BLAST-search or literature review, and will be further investigated. Details on the repertoire of resistance genes and associated ICEs for each individual isolate is detailed in Supplementary Table S1.

## Discussion

In the present study, we have conducted a comprehensive analysis of macrolide and clindamycin resistance in invasive BHS isolates over a 15-year period, and to the best of our knowledge, this is the first study investigating the temporal trends of such resistance in SD isolates.

Alarmingly, we found significantly increasing resistance rates in our health region for SD, rising incrementally from the start of the current decade. Whole genome sequencing of the resistant bacterial population revealed a substantial phylogenetic diversity, but the mobile genetic elements harboring the resistance genes displayed a high degree of genetic similarity. Taken together this indicates dissemination of MLS_*B*_-resistance genes in SD predominantly through conjugative transfer rather than clonal dissemination.

Although several studies have described a high diversity of *emm*-types among resistant SD isolates, knowledge on MLST-profiles in this population is scarce (Wajima et al., 2016; Zheng et al., 2017; Kim et al., 2018). Kim et al. (2018) recently characterized contemporary SD isolates from Japan and Korea, and reported a polyclonal distribution, with 13 *emm*/ST profiles among 20 isolates and 6 *emm*/ST profiles among 24 isolates in the two countries, respectively. Strikingly, despite the temporal and geographic proximity they only detected two mutual resistant clones, *stG485*/ST128/*mef*(A) and *stG840*/ST269/*erm*(B). Those clones were also present in our community, but no further similarities were evident. This underlines the highly polyclonal distribution of SD isolates, but could also infer the existence of some globally successful lineages. However, the paucity of data precludes firm conclusions.

Conversely, we found erythromycin and clindamycin resistance in GAS to be almost exclusively linked to a transient outbreak of an *emm11*/ST403/*erm*(B) lineage during 2010–2012. The emergence of this lineage has previously been documented in several other countries, though a few years prior to its arrival in Norway (Perez-Trallero et al., 2007; Silva-Costa et al., 2008). Although such clonal outbreaks can have a rapid and major impact on resistance rates, surging from 2 to 20% in 1 year in our region, the long-term implications are often negligible. With the gradual acquisition of specific immunity in the general population, the outbreak usually subsides (Montes et al., 2014).

Substantiating this, longitudinal epidemiologic surveillance in Spain and Portugal from the mid-90s revealed the erythromycin resistant population in GAS to be highly clonal (Perez-Trallero et al., 2007; Silva-Costa et al., 2012; Montes et al., 2014). The emergence and disappearance of new dominant clones continuously renders the phylogenetic landscape and is reflected in the fluctuating course of the resistance rates (Silva-Costa et al., 2008; Montes et al., 2014). From erythromycin resistance rates approximating 30% in the late 90s, the problem has almost vanished, and is currently below 5% in Portugal (Friaes et al., 2019). National surveillance reports from the Nordic countries during the past five years have all documented low resistance rates to MLS_*B*_ antibiotics in GAS, indicating that the emergence of new resistant GAS clones is a rare event in northern Europe (DANMAP, 2017; Norm/Norm-Vet, 2018; Swedres-Swarm, 2018).

In the present study, a significant upward trend for resistance to erythromycin and clindamycin was detected in GBS, exceeding 20% for both agents by the end of the study period. A similar development and level of resistance has been documented in our neighboring countries (DANMAP, 2017; Public Health England, 2018; Swedres-Swarm, 2018). The United States of America appeared to start down this path some years earlier, and the resistance rates approximated 50% by 2015^5^.

Like in SD, the gradually increasing resistance to erythromycin and clindamycin in GBS in our region was predominantly caused by a polyclonal expansion of the resistant population. A comparable diversity has been reported by others, including a similar distribution of capsule-types and MLST-profiles (Bergseng et al., 2008; Rojo-Bezares et al., 2016). However, given the limited number of capsule-types as compared to GAS and SD *emm*-types, the genotype/ST-profile/resistance-gene annotation provides a much lower phylogenetic resolution in GBS, making cross-study comparisons more difficult.

The past decades SD has emerged as an important cause of human infectious disease, and in Finland, it was the fifth most frequent pathogen detected in blood cultures in 2016 (Finland National Institute for Health and Welfare, 2016). Whereas GAS and GBS are under national surveillance in several countries, almost none monitor the epidemiology of SD on a national level. Public Health England report annual resistance rates for BHS, but present data for group C and group G streptococci separately, without identification to the species level (Public Health England, 2018). Furthermore, susceptibility data is only available for 60–70% of the isolates, posing a risk of reporting bias. Nonetheless, the major increase in resistance observed in the United Kingdom over the past decades is alarming, rising from 13 to 40% and 7 to 30% in group G streptococci for erythromycin and clindamycin, respectively. Moreover, even higher rates have been reported from Asia (Zheng et al., 2017). Although the level of resistance in SD in our community is still low in this context, the general direction of the temporal trend along with the polyclonal nature of the resistant isolates are concerning. Antimicrobial resistance spreading by conjugative transfer is less affected by herd immunity in the host population, and thus likely more challenging to combat (Lerminiaux and Cameron, 2019).

The *erm*(A) carrying integrative mobilizable element IME*sp2907* was initially discovered in GAS as a part of a larger composite ICE*sp2905*, also harboring a separate Tet(O) element (Giovanetti et al., 2012). All these modules could be transferred independently or collectively to new GAS recipients, revealing the intricate yet highly versatile transfer dynamics of horizontal genetic exchange between streptococcal species. Interestingly, we found homologs of IME*sp2907* in all three species of BHS, but in varying genomic contexts. On a similar note, the distribution of the different resistance determinant associated ICEs in *S. agalactiae* and *S. dysgalactiae* did not appear to intimately correlate with phylogenetic origin as judged by core-genome SNP-analysis. Taken together, this could infer a pivotal role for conjugative transfer in the dissemination of antimicrobial resistance among BHS. Furthermore, by searching the NCBI database, an IME*sp2907* homolog can also be detected in *Streptococcus anginosus* and *Anaerococcus* species, raising the question of the origin of these genetic elements. Hence, the emphasis of future research on resistance determinants in BHS should be to identify their major reservoirs, and the investigate the factors that influence the frequency of conjugative spread.

This study is limited by the low number of resistant isolates. However, in 2018 all three BHS species were included in the Norwegian Surveillance Program for Antimicrobial Resistance (NORM) for the first time, confirming that our findings reflect the national incidence of resistance to erythromycin and clindamycin (Norm/Norm-Vet, 2018). The transferability of our data to regions of high-level resistance remains to be elucidated, and further molecular studies on the resistant BHS population are warranted, especially for SD.

## Conclusion

Resistance to erythromycin and clindamycin in GBS and SD is increasing in western Norway. The resistant bacterial population is highly polyclonal, but a limited number of ICEs appear to mediate the dissemination of resistance genes. This could indicate a spread through conjugal transfer rather than clonal expansion, but further studies are needed to elucidate the mechanisms of transmission.

## Data Availability Statement

Whole genome data for all sequenced isolates has been deposited at DDBJ/ENA/GenBank under the Bioproject PRJNA600655, and the NCBI accession numbers JAAABO000000000–JAAAEW000000000.

## Author Contributions

OO and BK conceived the study. SS and HM contributed to the study design. OO performed statistical analysis and bioinformatics, and drafted the manuscript. All authors read and approved the final manuscript.

## Conflict of Interest

The authors declare that the research was conducted in the absence of any commercial or financial relationships that could be construed as a potential conflict of interest.

## Footnotes

1 wwwn.cdc.gov/BactFacts

2 www.Eucast.org

3 https://www.bioinformatics.babraham.ac.uk/projects/fastqc

4 https://www.cdc.gov/streplab/groupa-strep/

5 wwwn.cdc.gov/BactFacts

## Supplementary Material

TABLE S1 Overview of resistance genes and their associated mobile genetic elements.
